# Stem cell-based biological tooth repair and regeneration

**DOI:** 10.1016/j.tcb.2010.09.012

**Published:** 2010-12

**Authors:** Ana Angelova Volponi, Yvonne Pang, Paul T. Sharpe

**Affiliations:** 1 Department of Craniofacial Development and MRC Centre for Transplantation, Kings College London; NIHR comprehensive Biomedical Research Centre at Guys and St Thomas’ NHS Foundation Trust and Kings College London, London, UK; 2 Advanced Centre for Biochemical Engineering, University College London, London, UK

## Abstract

Teeth exhibit limited repair in response to damage, and dental pulp stem cells probably provide a source of cells to replace those damaged and to facilitate repair. Stem cells in other parts of the tooth, such as the periodontal ligament and growing roots, play more dynamic roles in tooth function and development. Dental stem cells can be obtained with ease, making them an attractive source of autologous stem cells for use in restoring vital pulp tissue removed because of infection, in regeneration of periodontal ligament lost in periodontal disease, and for generation of complete or partial tooth structures to form biological implants. As dental stem cells share properties with mesenchymal stem cells, there is also considerable interest in their wider potential to treat disorders involving mesenchymal (or indeed non-mesenchymal) cell derivatives, such as in Parkinson's disease.

## Tooth development

Teeth are complex organs containing two separate specialized hard tissues, dentine and enamel, which form an integrated attachment complex with bone via a specialized (periodontal) ligament. Embryologically, teeth are ectodermal organs that form from sequential reciprocal interactions between oral epithelial cells (ectoderm) and cranial neural crest derived mesenchymal cells. The epithelial cells give rise to enamel forming ameloblasts, and the mesenchymal cells form all other differentiated cells (e.g., dentine forming odontoblasts, pulp, periodontal ligament) (Box 1). Teeth continue developing postnatally; the outer covering of enamel gradually becomes harder, and root formation, which is essential for tooth function, only starts to occur as part of tooth eruption in children.

Repair, restoration and replacement of teeth is unique among clinical treatments because of the huge numbers of patients involved. Paradoxically, although teeth are nonessential for life and thus not considered a major target for regenerative medicine research, in comparison with neural or cardiac diseases, for example, this very fact makes teeth ideal for testing new cell-based treatments. Because the patients are not usually ill, if anything goes wrong it is far less life threatening, and the accessibility of teeth means that treatment does not require major surgery. Added to this is the existence of highly proliferative stem cell populations in teeth, which can be easily obtained from naturally lost or surgically removed teeth. These stem cells can be used for tooth repair, restoration and regeneration and, significantly, non-dental uses, such as developing stem cell-based therapies for major life-threatening diseases. An important but often overlooked advantage of teeth as a source of stem cells is that postnatal root formation (a rich source of dental stem cells) is a developmental process, and thus cells involved in root formation are more embryonic-like than other sources of dental stem cells. The humble tooth clearly has a very important role to play in future developments in regenerative medicine.

In this review, we outline the important biological properties of dental stem cells and illustrate examples of research showing the rapid progress being made in using these cells for tooth repair. We also highlight the major obstacles that need to be overcome before any form of usable, cell-based tooth replacement becomes available to practising dentists.

## Dental stem cells

Several populations of cells with stem cell properties have been isolated from different parts of the tooth. These include cells from the pulp of both exfoliated (children's) and adult teeth, from the periodontal ligament that links the tooth root with the bone, from the tips of developing roots and from the tissue (dental follicle) that surrounds the unerupted tooth. All these cells probably share a common lineage of being derived from neural crest cells and all have generic mesenchymal stem cell-like properties, including expression of marker genes and differentiation into mesenchymal cell lineages (osteoblasts, chondrocytes and adipocytes) *in vitro* and, to some extent, *in vivo*. The different cell populations do, however, differ in certain aspects of their growth rate in culture, marker gene expression and cell differentiation, although the extent to which these differences can be attributed to tissue of origin, function or culture conditions remains unclear.

## Dental pulp stem cells

The possibility that tooth pulp might contain mesenchymal stem cells was first suggested by the observation that severe tooth damage that penetrates both enamel and dentine and into the pulp stimulates a limited natural repair process, by which new odontoblasts are formed, which produce new dentine to repair the lesion [1,2]. Putative stem cells from the tooth pulp and several other dental tissues have now been identified (Box 2) [3–8].

The first stem cells isolated from adult human dental pulp were termed dental pulp stem cells (DPSCs) [3]. They were isolated from permanent third molars, and exhibited high proliferation and high frequency of colony formation that produced sporadic, but densely calcified nodules. Additionally, *in vivo* transplantation into immunocompromised mice demonstrated the ability of DPSCs to generate functional dental tissue in the form of dentine/pulp-like complexes [4]. Further characterization revealed that DPSCs were also capable of differentiating into other mesenchymal cell derivatives *in vitro* such as odontoblasts, adipoctyes, chondrocytes and osteoblasts [9–12]. DPSCs differentiate into functionally active neurons, and implanted DPSCs induce endogenous axon guidance, suggesting their potential as cellular therapy for neuronal disorders [13–15].

## Stem cells from human exfoliated deciduous teeth

Stem cells isolated from the pulp of human exfoliated deciduous (children's milk) teeth (SHED) have the capacity to induce bone formation, generate dentine and differentiate into other non-dental mesenchymal cell derivatives *in vitro* [16–20]. In contrast to DPSCs, SHED exhibit higher proliferation rates [21], increased population doublings, osteoinductive capacity *in vivo* and an ability to form sphere-like clusters [16]. SHED seeded onto tooth slices/scaffolds and implanted subcutaneously into immunodeficient mice differentiated into functional odontoblasts capable of generating tubular dentine and angiogenic endothelial cells [18].

Studies using SHED as a tool in dental pulp tissue engineering *in vivo,* where pulp removed because of infection is replaced with stem cells, have revealed that the tissue formed has architecture and cellularity closely resembling that of dental pulp, a tissue important for tooth vitality [19]. Another interesting clinical application has been suggested by investigations of the therapeutic efficacy of SHED in alleviating Parkinson's disease (PD) [20]. Transplantation of SHED spheres into the striatum of parkinsonian rats partially improved the apomorphine evoked rotation of behavioural disorders. The results of this study indicate that SHED might be a useful source of postnatal stem cells for PD treatment. SHED are isolated from children's exfoliated teeth, however, so autologous stem cell therapy for a disease such as PD would require that these cells be stored from childhood. DPSCs, which are obtained from adult tooth pulp, might well have similar properties, however, and collection and expansion of these autologous cells would simply require removal of a tooth from the patient.

SHED and other dental stem cells are derived from cranial neural crest ectomesenchyme, and so developmentally and functionally would appear identical, but studies have shown that they do differ and have different gene expression profiles. SHED have significantly higher proliferation rates compared with DPSC and bone marrow-derived mesenchymal stem cells [21]. Comparison of the gene expression profiles showed 4386 genes that are differentially expressed between DPSC and SHED by two-fold or more. Higher expression in SHED was observed for genes that participate in pathways related to cell proliferation and extracellular matrix formation, including several growth factors such as fibroblast growth factor and transforming growth factor (TGF)-β [21]. TGF-β in particular is important, because it is released after damage to dentine and might act to mobilize pulp stem cells to differentiate into odontoblasts [1,22].

DPSC are highly proliferative and retain their stem cell characteristics after prolonged culture [23]. They could therefore be used as a generic allogeneic source of mesenchymal stem cells. Their use as autologous cells, however, is currently restricted to children who have not yet lost all their deciduous teeth. Commercial banking of these cells is thus becoming widespread to enable them to be used once the child becomes an adult. Limited studies have shown that frozen SHED cells do maintain their properties after cryopreservation for 2 years [24], but one caveat is that the effects of long-term storage (10 years, plus) have not yet been assessed. Because children naturally lose 20 deciduous teeth, there are multiple opportunities to bank these cells, unlike cord blood, for example.

## Periodontal ligament stem cells

The periodontal ligament (PDL) is a fibrous connective tissue that contains specialized cells located between the bone-like cementum and the inner wall of the alveolar bone socket that acts as a ‘shock absorber’ during mastication (Box 2). The PDL has long been recognized to contain a population of progenitor cells [25] and recently, several studies [26] identified a population of stem cells from human periodontal ligament (PDLSC) capable of differentiating along mesenchymal cell lineages to produce cementoblast-like cells, adipocytes and connective tissue rich in collagen I *in vitro* and *in vivo* [26–29].

The periodontal ligament is under constant strain from the forces of mastication, and thus PDLSC are likely to play an endogenous role in maintaining PDL cell numbers. This might explain why they are better than other dental stem cell populations at forming PDL-like structures [17].

## Root apical papilla stem cells

A unique population of dental stem cells known as stem cells from the root apical papilla (SCAP) is located at the tips of growing tooth roots (Box 2). The apical papilla tissue is only present during root development before the tooth erupts into the oral cavity [30]. SCAP have the capacity to differentiate into odontoblasts and adipocytes [27]. These cells are CD24+ but expression is downregulated upon odontogenic differentiation *in vitro* coincident with alkaline phosphatase upregulation. SCAP cells exhibit higher rates of proliferation *in vitro* than do DPSC [27]. By co-transplanting SCAP cells (to form a root) and PDLSC (to form a periodontal ligament) into tooth sockets of mini pigs, dentine and periodontal ligament was formed. These findings suggest that this population of cells, together with PDLSC, could be used to create a biological root that could be used in a similar way as a metal implant, by capping with an artificial dental crown. Most human tissues from early in their development are not clinically available for stem cell isolation; however, because roots develop postnatally, the root apical papilla is accessible in dental clinical practice from extracted wisdom teeth. Thus, a very active source of stem cells with embryonic-like properties (i.e., in the process of development) can be readily obtained. Further experiments on the properties of these cells obtained from human teeth following expansion in culture are needed.

## Dental follicle stem cells

The dental follicle is a loose ectomesenchyme-derived connective tissue sac surrounding the enamel organ and the dental papilla of the developing tooth germ before eruption [31]. It is believed to contain progenitors for cementoblasts, PDL and osteoblasts. Dental follicle cells (DFC) form the PDL by differentiating into PDL fibroblasts that secrete collagen and interact with fibres on the surfaces of adjacent bone and cementum. DFC can form cementoblast-like cells after transplantation into SCID mice [32,33].

Dental follicle progenitor cells isolated from human third molars are characterized by their rapid attachment in culture, expression of the putative stem cell markers Nestin and Notch-1, and ability to form compact calcified nodules *in vitro* [34]. When DFC were transplanted into immunocompromised mice, however, there was little indication of cementum or bone formation [34]. DFC, in common with SCAP, represent cells from a developing tissue and might thus exhibit a greater plasticity than other dental stem cells. However, also similar to SCAP, further research needs to be carried out on the properties and potential uses of these cells.

## Dental tissue repair

There are several areas of research for which dental stem cells are currently considered to offer potential for tissue regeneration. These include the obvious uses of cells to repair damaged tooth tissues such as dentine, periodontal ligament and dental pulp [16–19,32–36]. Even enamel tissue engineering has been suggested [37], as well as the use of dental stem cells as sources of cells to facilitate repair of non-dental tissues such as bone and nerves [12–15,20,38,39].

## Periodontal regeneration

The periodontium is a set of specialized tissues that surround and support the teeth to maintain them in the jaw. Periodontitis is an inflammatory disease that affects the periodontium and results in irreversible loss of connective tissue attachment and the supporting alveolar bone. The challenge for cell-based replacement of a functional periodontium is therefore to form new ligament and bone, and to ensure that the appropriate connections are made between these tissues, as well as between the bone and tooth root. This is not a trivial undertaking, as these are very different tissues that form in an ordered manner (spatially and temporally) during tooth development. One aim of current research is to use different populations of dental stem cells to replicate the key events in periodontal development both temporally and spatially, so that healing can occur in a sequential manner to regenerate the periodontium [34].

A conceptually simpler approach to periodontal regeneration methods involves engineered cell sheets to facilitate human periodontal ligament (HPDL) cell transplantation [35]. Periodontal ligament cells isolated from a human third molar tooth were cultured on poly(*N*-isopropylacryl-amide) (PIPAAm)-grafted dishes that induce spontaneous detachment of the cells as viable cell sheets upon low temperature treatment. HPDL cells sheets were implanted into athymic rats that had the periodontium and cementum removed from their first molars. Fibril anchoring resembling native periodontal ligament fibres, together with an acellular cementum-like layer, was observed, indicating that this technique could be applicable to future periodontal regeneration. Although promising, this approach does not take into account any replacement of bone that might be required.

The outstanding issue with these approaches is the extent to which any reconstituted periodontium can maintain integrity and function during mastication over long periods of time. Current treatments for severe periodontitis are poor, however, and thus, despite their flaws, any new dental stem cell-based treatments are likely to be the subject of intensive clinical research in the near future.

## *De novo* regeneration of dental pulp

Dental pulp needs to be removed when it becomes infected, and this is particularly problematic for root pulp that requires endodontic (root canal) treatment. The restoration of tooth pulp is thus a much sought after goal in dentistry because the current practice of replacing infected pulp with inorganic materials (cements) results in a devitalized (dead) tooth. A recent study demonstrated *de novo* regeneration of dental pulp in emptied root canal space using dental stem cells [36]. DPSC and SCAP isolated from the human third molars were seeded onto a poly-D,L-lactide/glycolide scaffold and inserted into the canal space of root fragments, followed by subcutaneous transplantation into SCID mice. Subsequent histological analysis of the tooth fragments 3–4 months after surgery indicated that the root canal space was completely filled with pulp-like tissue with well established vascularization. Moreover, a continuous layer of mineralized tissue resembling dentine was deposited on the existing dentinal walls of the canal [36]. Recent studies using genetically marked cells in mice have suggested that adding stem cells makes little difference to the extent to which an empty pulp cavity regenerates because the majority of cells are provided by the vasculature (Sharpe P.T, unpublished data). Stem cell pulp restoration might therefore not be a problem of providing exogenous stem cells but one of surgically ensuring that an adequate blood supply is maintained after pulp removal.

## Whole tooth regeneration

The current state of the art in tooth replacement is a dental implant that involves screwing a threaded metal rod into a predrilled hole in the bone, which is then capped with a plastic or ceramic crown. Implant use requires a minimum amount of bone to be present. Because these implants attach directly to the bone without the PDL ‘shock absorber’, the forces of mastication are transmitted directly to the bone, which is one reason implants can fail. In cases where there is insufficient bone for implants, such as tooth loss as a consequence of the bone loss that occurs in postmenopausal osteoporosis, implants have to be preceded by bone grafts. The ultimate goal in dentistry is to have a method to biologically replace lost teeth; in essence, a cell-based implant rather than a metal one. The minimum requirement for a biological replacement is to form the essential components required for a functional tooth, including roots, periodontal ligament, and nerve and blood supplies. Paradoxically, the visible part of the tooth, the crown, is less important because, although essential for function, synthetic tooth crowns function well, and can be perfectly matched for size, shape and colour. The challenge, therefore, for biological tooth replacement is ultimately one of forming a biological root.

**Figure 1:**
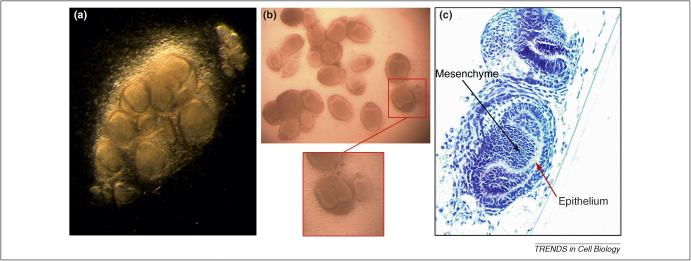
Tooth formation *in vitro* from combinations of mouse epithelial and mesenchymal cells. The epithelium (red arrow) and mesenchyme (black arrow) are separated from pre-bud stage tooth primordia,and cells dissociated in single cell populations. **(a)** The cells are recombined (as shown in this figure) and grown *in vitro* for 6 days. **(b)** Gross appearance after 9 days in culture with higher magnification of a tooth primordium. **(c)** Sections of tooth primordia from **(a)**, showing development to the bell stage.

Currently, the major challenges in whole tooth regeneration are to identify non-embryonic sources of cells with the same properties as tooth germ cells and to develop culture systems that can expand cells that retain tooth forming potential (Figure 1). This is even more challenging when considering the fact that tooth development requires two cell types, epithelial and mesenchymal [40–42].

The induction of odontogenic potential lies in the dental epithelium [43–45]. Dental epithelium from pre-bud stages can induce tooth formation when combined with nonodontogenic mesenchyme as long as the mesenchymal cells have stem cell-like properties in common with neural crest cells [46]. After epithelial induction of the mesenchyme, this becomes the inductive tissue and reciprocates inductive signals back to the now noninductive epithelium. Tooth regeneration can thus be approached in one of two ways; identification of either epithelial or mesenchymal cells than can induce tooth formation in the other cell type.

## Odontogenic responsive epithelial cells

No sources of epithelial cells capable of inducing odontogenesis have been identified to date, other than the endogenous dental epithelium of early stage embryos. The main limitation for identifying sources of epithelial cells that can be grown in culture and form teeth after association with inducing mesenchymal cells is that these epithelial cells retain an immature state.

The epithelial rests of Malassez (ERM) are a group of cells that remain during root formation; thus, these cells are present in adult teeth and can be isolated and cultured [51–55]. When ERM cells are maintained *in vitro* on feeder layers, they can be induced to form enamel-like tissues following recombination with primary (uncultured) dental pulp cells [55].

Oral mucosa epithelial cells from embryos and adults have been used in recombination experiments and shown to give rise to complex dental structures, but not whole functional teeth, when combined with embryonic dental mesenchyme [56,57]. Some evidence of tooth formation was seen when oral epithelial lines established from p-53-deficient mouse embryos at embryonic day (E)18 were combined with fetal E16.5 molar mesenchymal tissues and transplanted for 2–3 weeks [56]. Postnatal oral mucosal epithelium might also offer some potential as a replacement for embryonic dental epithelium, because cells isolated from young animals, grown as cell sheets and re-associated with dental mesenchyme from E12.5 embryos, can give rise to tooth-like structures [57].

There are sources of epithelial cells that can contribute to tooth formation following culture, suggesting that exogenously adding factors to these cells could make them inducible. Such factors, include signalling proteins of the fibroblast growth factor bone morphogenetic protein and Wnt families, but the issue of reproducing the temporal, spatial and quantitative delivery of these, as seen *in vivo*, is daunting. Identification of key intracellular factors (e.g., kinases, transcription factors.) is likely to be a more fruitful direction because these are more easily manipulated.

## Odontogenic inductive mesenchymal cells

The ability of non-dental mesenchymal cell sources to respond to odontogenic epithelial signals following *in vitro* expansion was demonstrated when it was shown that expanded adult bone marrow stromal cells would form teeth *in vitro* when combined with inductive embryonic oral epithelium [46]. This study also showed that embryonic tooth primordia could develop into complete teeth, following transplantation into the adult oral cavity. Such transplants, when left for sufficient time, will form roots and erupt [47,58]. The issues with producing inductive epithelium *in vitro* illustrated in the above section suggest that the alternative approach of identifying mesenchymal cells with inductive capacity might be more fruitful. The cells that have this capacity *in vivo* are the early embryonic neural crest-derived ectomesenchyme cells that have already received the first inductive signals from the dental epithelium (Box 1). Bone marrow mesenchymal cells, although able to respond to odontogenic signals from the epithelium, are only able to induce tooth formation after receiving these epithelial signals. Such priming of bone marrow mesenchymal cells by inducing factors or embryonic dental epithelium is possible, but in reality too laborious and difficult to be of any clinical value.

If ectomesenchyme cells have odontogenic-inducing capacity, can this be maintained *in vitro*? Embryonic tooth primordia mesenchymal cells from mice have been shown to retain their potential to respond to odontogenic signals following *in vitro* culture after immortalization but it is uncertain if cells with inducing capacity can retain this following culture (Jung H.-S., personal communication). Similarly, equivalent cells from human embryos have been isolated and shown to form teeth in re-association experiments (Volponi A.A. and Sharpe P.T., unpublished data).

Adult dental pulp mesenchymal stem cells are an obvious source of cells to replace embryonic ectomesenchyme because they are derived from cranial neural crest and are ‘dental’ cells. Indeed, these cells retain expression of many genes expressed in neural crest, in addition to a number of stem cell ‘marker’ genes. However, it has yet to be shown that adult dental pulp mesenchymal stem cells retain any odontogenic inductive or responsive capacity. One interesting direction is to identify the factors expressed by ectomesenchyme cells (embryonic dental mesenchyme) that render them capable of forming teeth that are not expressed by adult dental stem cells. Approaches similar to those developed for producing induced pluripotent stem cells can be used to convert adult dental stem cells into ectomesenchyme cells that can form teeth.

## Tooth regeneration by cell re-aggregation

**Figure 2:**
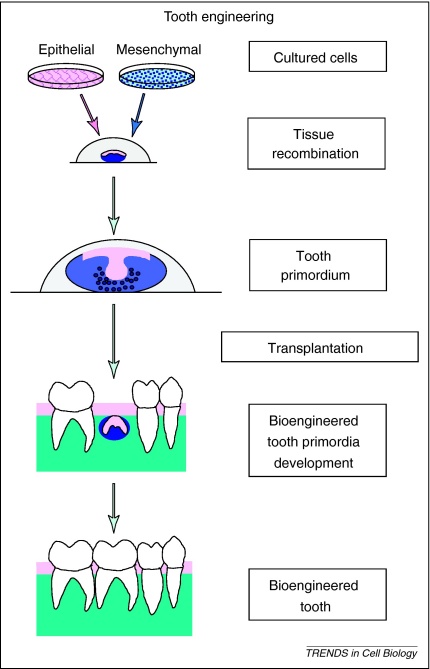
Diagrammatic representation of the generation of biological replacement teeth. Suitable sources of epithelial and mesenchymal cells are expanded in culture to generate sufficient cells. The two cell populations are combined to bring the epithelial and mesenchymal cells into direct contact, mimicking the *in vivo* arrangement. Interaction between these cell types leads to formation of an early stage tooth primordium, equivalent to a tooth bud or cap, around which the mesenchyme cells condense (dark blue dots) (see also Box 1). The tooth primordium is surgically transplanted into the mouth and left to develop.

Functional teeth can be experimentally bioengineered in mice by re-association of dissociated tooth cells [48–51]. These experiments actually demonstrate the ability of dissociated cells to re-aggregate, however, rather than the bioengineering of whole teeth. The cells used are obtained from embryonic tooth primordia, many of which are required to produce one tooth. When tooth germs are dissociated and allowed to re-associate in an extracellular matrix (scaffold), they ‘sort out’ and re-aggregate to reform the tooth germs [48,51]. The re-aggregation produces multiple small ‘toothlets’, whose shape bears no resemblance to that of the scaffold used (Figures 1 and 2). Similarly, the tooth germ epithelial and mesenchymal cell components can be physically separated, the cells dissociated and recombined, whereupon they ‘sort’ and re-aggregate to reform the tooth germ [48,51]. In this case, 5 × 10^4^ cells dissociated from multiple tooth germs are required to generate a single new tooth germ [48]. The large cell numbers required necessitate *in vitro* expansion of epithelial and mesenchymal cells that will retain their odontogenic properties.

## Concluding remarks

Despite some progress, there remain major obstacles to formulating safe, simple and reproducible cell-based approaches for tooth repair and regeneration that could be used on patients. It is clear that there is both a clinical need for such treatments and a vast patient resource. Dental stem cells have many advantages, and results to date suggest that teeth are a viable source of adult mesenchymal stem cells for a wide range of clinical applications. Ultimately, the use of these dental stem cells over other sources of mesenchymal stem cells for therapeutic use will not only depend on ease of use and accessibility, but also on the efficiency and quality of repair in relation to cost. Dental pulp cells grow well in culture and, unusually, the proportion of cells with stem cell properties appears to increase with passage. The molecular basis of this phenomenon needs to be investigated because it might provide a paradigm for increasing stem cell numbers in cultures of other cell types.

For whole tooth regeneration, there remain many major issues that will take considerable time to resolve. Most immediate is the identification of epithelial and mesenchymal cell populations that can be maintained and expanded in culture to provide the large numbers needed to make a tooth. Related to this is the issue of whether the cells will need to be autologous (expensive, but safe) or allogeneic (cheaper, but with possible rejection problems). Finally, an additional fundamental issue that needs to be considered is that human tooth development is a much slower process than in mice. Human tooth embryogenesis is approximately eight times slower, and postnatal development lasts several years. Thus, whereas growth, implantation and eruption of bioengineered mouse teeth might take a few weeks, the equivalent time to create a functional human tooth might be many months or even years. Research thus needs to be done to investigate ways of possibly accelerating human tooth development.

## Figures and Tables

**Figure I:**
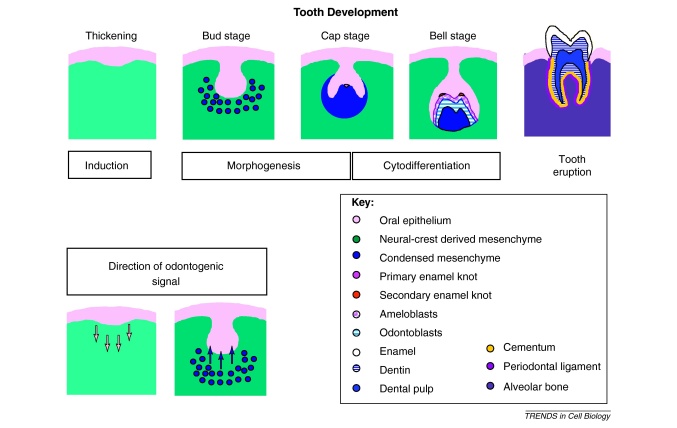
Diagrammatic representation of tooth development.

**Figure II:**
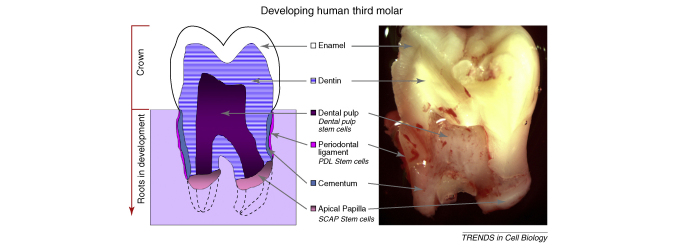
**Photograph and diagram of a human third molar tooth following extraction**. A hemisected tooth showing the internal tissues is shown on the right. Because the tooth was in the process of erupting, root growth is incomplete, and the apical papilla is visible. A diagrammatic representation of this tooth is shown on the left.

## Box 1 Tooth development

Tooth development is traditionally considered a series of stages that reflect key processes (Figure I). The first step is induction, in which signals from the epithelium to the mesenchyme initiate the developmental process. As localized proliferation of the dental epithelial cells takes place, the cells form a bud around which the mesenchymal cells condense. Differentiation and localized proliferation of the epithelial cells in the bud leads to the cap stage. It is at this stage that crown morphogenesis is initiated by the epithelial signalling centre, an enamel knot regulating the folding of the epithelium. By the bell stage, the precursors of the specialized tooth cells, ameloblasts, coordinate enamel deposition, and odontoblasts, which produce dentine, are formed. Tooth eruption involves the coordination of bone resorption and root development, and occurs postnatally.

Throughout tooth development, signals are exchanged between epithelial and mesenchymal cells to coordinate each process. The key initial signals occur at induction (epithelium) and bud formation (mesenchyme). Once the mesenchymal cells receive signals from the epithelium, the mesenchyme sends reciprocal signals back to the epithelium. Strategies for biological replacement teeth aim to utilize these first signal exchanges by identifying either epithelial cells that can induce a naive mesenchyme or mesenchymal cells that can induce a naive epithelium to stimulate tooth development.

## Box 2 Human third molar as a source of dental stem cells

Human third molars (‘wisdom teeth’) start their development postnatally, during childhood (ages of 5–6 years) and begin their calcification process from the age of 7–10 years. By the age of 18–25 years, the roots of the third molars have completed their development. These teeth are most commonly extracted and discarded in the dental clinic, but because they are still undergoing root development, they provide an excellent source of dental stem cells including DPSC, PDL cells and SCAP cells (Figure II).
